# Gender-Related Differences in the Rates of Age Associated Thymic Atrophy

**DOI:** 10.1155/2001/17064

**Published:** 2001

**Authors:** Richard Aspinall, Deborah Andrew

**Affiliations:** Department of Immunology Imperial College of Science Technology and Medicine Chelsea & Westminster Hospital 369 Fulham Road London SW10 9NH UK

## Abstract

Age associated thymic atrophy has been shown to be linked to problems with rearrangement of the β chain of the T cell receptor (TCR) in male mice during the early phases of the intrathymic T cell developmental pathway. In this study, thymic atrophy in female mice was found to occur at a different rate than in male mice. At 9 months of age there was a significantly greater number of cells in the thymus of female mice compared with male mice, with the major difference found in the CD4^+^CD8^+^ populations. The thymii of female mice at 9 months of age contained double the number of these cells compared with male mice. Analysis of the CD4^+^CD8^+^ cells at 9 months of age demonstrated increased numbers of cells expressing higher levels of CD3 in females compared with males indicating that in females more of these cells were producing successful αβTCR pairings. In F5 transgenic mice comparison of the CD4^+^CD8^+^ population revealed no significant difference in their absolute numbers at 9 months of age. These results indicate that the gender differences at this time point were due to fewer permitted divisions prior to the expression of a selectable TCR α chain within the CD4^+^CD8^+^ populations in male compared with female mice. This gender difference was not due to the action of testosterone and unlikely to be due to differences in the level of oestrogen. The potential mechanisms of this difference may be related to a regulatory feedback of peripheral T cells on the developing thymocyte populations. Such age related changes in the numbers of cells within distinct thymic subpopulations leads to the possibility that the potential repertoire in females is greater than in males later in life.


*Developmental Immunology*, 2001, Vol. 8(2), pp. 95-106
Reprints available directly from the publisher
Photocopying permitted by license only

© 2001 OPA (Overseas Publishers Association)
N.V. Published by license under
the Harwood Academic Publishers imprint,
part of The Gordon and Breach Publishing Group,
a member of the Taylor and Francis Group

### Gender-Related Differences in the Rates of Age Associated Thymic Atrophy

RICHARD ASPINALL* and DEBORAH ANDREW

*Department of Immunology Imperial College of Science Technology and Medicine Chelsea & Westminster Hospital 369 Fulham Road London SW10 9NH*

Age associated thymic atrophy has been shown to be linked to problems with rearrangement of the β chain of the T cell receptor (TCR) in male mice during the early phases of the intrathymic T cell developmental pathway. In this study, thymic atrophy in female mice was found to occur at a different rate than in male mice. At 9 months of age there was a significantly greater number of cells in the thymus of female mice compared with male mice, with the major difference found in the $CD4^+CD8^+$ populations. The thymii of female mice at 9 months of age contained double the number of these cells compared with male mice. Analysis of the $CD4^+CD8^+$ cells at 9 months of age demonstrated increased numbers of cells expressing higher levels of CD3 in females compared with males indicating that in females more of these cells were producing successful αβTCR pairings. In F5 transgenic mice comparison of the $CD4^+CD8^+$ population revealed no significant difference in their absolute numbers at 9 months of age. These results indicate that the gender differences at this time point were due to fewer permitted divisions prior to the expression of a selectable TCR α chain within the $CD4^+CD8^+$ populations in male compared with female mice. This gender difference was not due to the action of testosterone and unlikely to be due to differences in the level of oestrogen. The potential mechanisms of this difference may be related to a regulatory feedback of peripheral T cells on the developing thymocyte populations. Such age related changes in the numbers of cells within distinct thymic subpopulations leads to the possibility that the potential repertoire in females is greater than in males later in life.

### INTRODUCTION

Age related alteration in the function of the immune system has been recognised in many species. The clinical presentations of such immune dysfunction are an age-related increased susceptibility to certain infections, an increased incidence of autoimmune disease, and certain cancers (Government Actuary's Department 1992, Office of National Statistics 1997, Gardner, 1980). Laboratory investigations reveal a reduced ability of the cells from older individuals, compared with younger individuals, to perform in functional *in vitro* assays (Flurkey et al., 1992; Bloom and Horvath, 1994). Furthermore, analysis of certain autoimmune diseases according to gender shows that more women than men are affected (Whitacre et al., 1999).

Such alterations in immune function may reflect changes in the peripheral T cell pool. Since there are no age-related changes either in the quantity of

* Proofs should be sent to Dr R. Aspinall, Department of Immunology Imperial College of Science Technology and Medicine Chelsea & Westminster Hospital 369 Fulham Road, London SW10 9NH, UK Tel: +44 (0)181 746 5993, FAX +44 (0)181 746 5997 e-mail r.aspinall@ic.ac.uk peripheral lymphocytes or in the number of $CD4^+$ or $CD8^+$ T lymphocytes (Hulstaert et al., 1994; Hannet et al., 1992) any difference must be related to the composition of these populations. This composition is affected by the thymic output since the rate at which the thymus produces T cells contributing to the peripheral T cell pool, declines with age (Mackall et al., 1995; Mackall and Gress, 1997).

The earliest precursors of the T cell pathway in the adult thymus are $CD3^-CD4^{lo}CD8^-$ (Wu et al., 1991). This population has been subdivided on the basis of expression of CD44 and CD25, with the most immature stage identified as $CD44^+CD25^-$. Differentiation from this stage involves the transient expression of CD25 and the gradual loss of CD44, during which time the β chain of the T cell receptor (TCR) is rearranged and expressed on the surface with an α chain equivalent (Godfrey et al., 1993; Godfrey et al., 1994). A productive receptor at this stage allows the cell to be positively selected and the resulting population is $CD44^-CD25^-CD3^-CD4^-CD8^-$ and differentiates to express both the CD4 and CD8 together. This immature thymocyte is the most abundant population in the thymus. During this phase the α chain undergoes rearrangement and expression and the resulting αβTCR binds to an MHC molecule containing a peptide on the epithelial cell (Jameson et al., 1995). The affinity of the interaction between the TCR and the selecting ligand determines the fate of the cell. Positive selection leads to down regulation of the RAG genes (Brandle et al., 1992) and progression to the next developmental stage. Failure leads to rearrangement and expression of another α chain and the formation of a new TCRαβ, a process repeated until the cell is either positively selected, or dies (Petrie et al., 1993). Positively selected cells progress to become mature thymocytes(Alam et al., 1996). These $CD4^+CD8^-$ or $CD4^-CD8^+$ cells are found in the medulla, have higher levels of expression of the TCR than their precursors, and many are cycling before they exit to the peripheral T cell pool (Penit and Vasseur, 1997).

Thymic involution has been studied recently in male mice and shown to be due to problems with rearrangement of the TCR-β chain genes affecting the production of thymocytes (Aspinall, 1997). Differences in the rate of human thymic involution between males and females has been described previously (Smith and Ossa Gomez, 1981; Simpson et al., 1975) although these descriptions have been contested (Steinmann, 1986). Here we report that thymic atrophy in normal C57BL/10 mice occurs earlier in males than in females resulting from a reduction in the number of $CD4^+CD8^+$ thymocytes prior to positive selection most significantly at 9 months of age. Female F5 transgenic mice, whose transgene is a complete TCRαβ specific for influenza nucleoprotein in the context of MHC $D^b$ inserted into a CD2 minigene cassette (Mamalaki et al., 1993), as in male F5 transgenic mice, show no significant age related thymic atrophy. The difference seen at 9 months of age between normal male and female mice was not seen between male and female F5 transgenic mice.

### MATERIALS AND METHODS

### Animals

Normal C57BL/10 mice were obtained from Harlan Olac (Oxfordshire, UK) and either placed in brother sister pairs to produce progeny, or maintained until used. F5 transgenic animals were obtained in the first instance from Dr D. Kioussis (National Institute of Medical Research, Mill Hill), and colonies of aging mice were derived by the brother sister mating of these original animals in positive pressure isolators in the animal house at ICSTM at Charing Cross.

### Derivation and Analysis of Tissue

Mice were killed by $CO_2$ asphyxiation. Thymuses were dissected cleanly out and thymocyte suspensions prepared as described previously (Aspinall, 1997). For the analysis of thymocyte subpopulations defined by their expression of CD4 and CD8, cells were stained and incubated with; biotin-conjugated anti-CD4 (clone KT6), streptavidin conjugated to Quantum Red, anti-CD3-FITC (clone 29B),

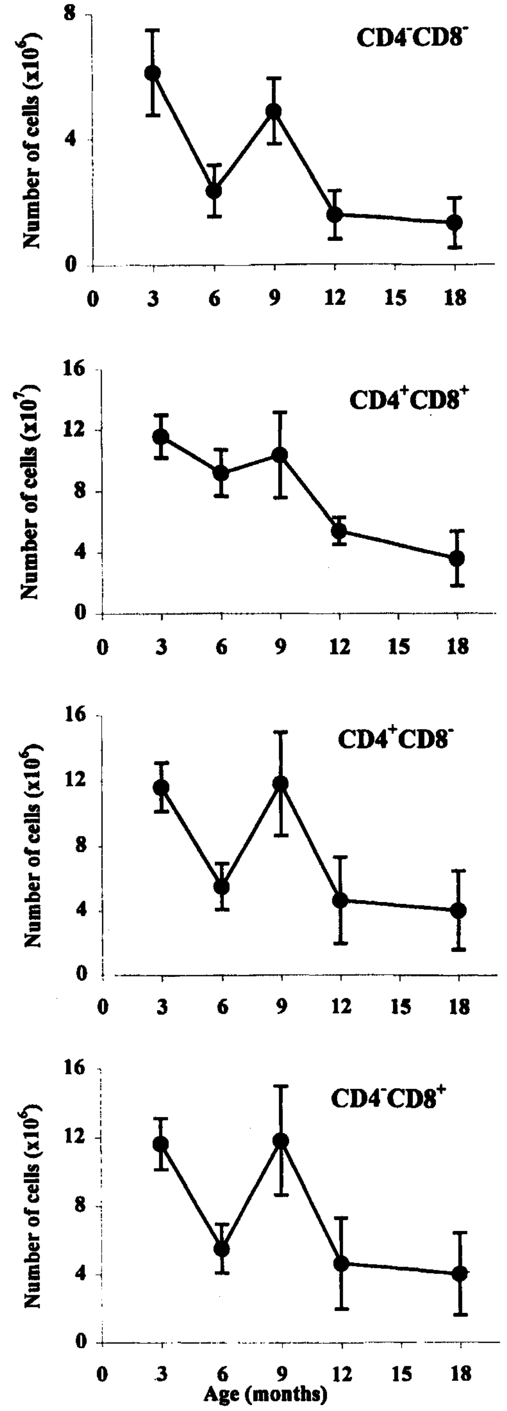

FIGURE 1 Analysis of female murine thymocyte populations defined by the expression of CD4 and CD8 demonstrate elevated levels at 9 months of age (n=3–7 animals at each time point). After this time there is a rapid decline in all subpopulations at 12 and 18 months of age anti-CD8a-PE (clone 53–6.7) and anti Vβ-11-FITC (clone KT 11). For the analysis of CD44 and CD25 expression on triple negative thymocytes, cells were stained with; biotin-conjugated anti-CD4 (clone KT6), biotin-conjugated anti-CD3 (clone KT 3), streptavidin conjugated to R- Phycoerythrin, anti-CD8-PE (clone 53–6.7), anti-CD44-cychrome™ (clone IM7), anti-CD25-FITC (clone7D4).

Control antibodies were conjugated to PE, FITC or biotin or cy-chrome™. Cells were fixed post-staining with 1% paraformaldehyde in PBS, and analysed on a Cytoron within 7 days of fixation, on a programme acquiring 20,000 (CD4 and CD8 expression) and 50,000 cells (CD25 and CD44 expression). The absolute number of cells in each subset was calculated from the percentage of positive cells of each subset multiplied by the total number of cells. All antibodies were obtained from Sigma UK Ltd, Serotec UK or Cambridge Biosciences (Pharmingen).

### Statistical Analysis

Statistical analysis was carried out using the unpaired 2 tailed T test with Microsoft Excel. Differences were considered significant for *P* value <0.05.

### RESULTS

### Age related changes in the female thymocyte subpopulations

Figure 1 shows the changes in the female thymic subpopulations with age. The most striking feature of changes in these thymic subsets is the high cell number in each subpopulation seen at 9 months of age. These changes are unlikely to be due to small sample sizes since at 6.5 months n=6 and at 9 months n=7. Comparison of all of the thymocyte subpopulations at 3 and 9 months of age shows no significant difference ($P$>0.05 for the $CD4^-CD8^-$, $CD4^+CD8^+$, $CD4^+CD8^-$ and $CD4^-CD8^+$ populations). By comparison the numbers of cells present at 6.5 months was significantly less than the number present at 3 months in the $CD4^-CD8^-$ ($P$<0.05), $CD4^+CD8^-$ ($P$<0.01) and $CD4^-CD8^+$ population ($P$<0.01). The $CD4^+CD8^+$ subpopulation shows only a marginally significant difference ($P$>0.05).

After 9 months of age there is a rapid decline in all subpopulations at 12 months and at 18 months of age with no significant difference seen in the numbers of cells in each subpopulation between 12 and 18 months of age (*P* was always greater than 0.05).

### Comparison of the numbers of thymocytes in male and female mouse thymuses

The decline in the numbers of cells within each thymic subset with age is similar in male and female mice such that at 6.5 months of age there is no significant difference in the total numbers within these thymic subsets ($P$>0.05). However analysis revealed that the rapid decline in total thymocyte cell number by 9 months of age, which is a characteristic in males was not present in females (Figure 2). At 9 months of age, and at no other time point, there is a significant difference in the total number of cells in the thymii of male and female mice ($P$<0.01).

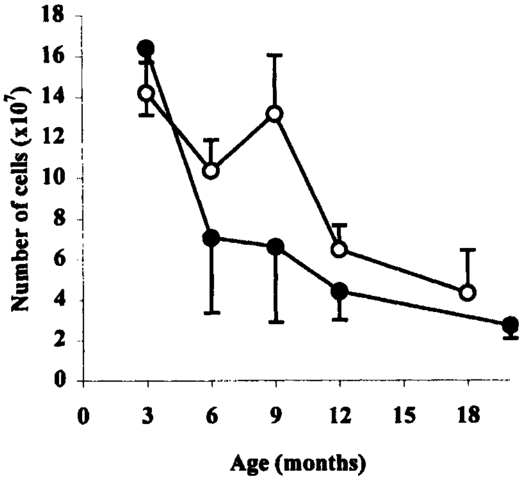

FIGURE 2 At 9 months of age and at no other time point there is a significant difference($P$<0.01) in the total number of cells in the thymus of male (filled circles) and female (unfilled circles) mice (n=3–7 animals at each time point)

### Comparison at 9 months of age

Analysis of the number of thymocytes within the $CD4^-CD8^-CD3^-$ population at 9 months of age are shown in Figure 3(i). These results reveal similar numbers of cells within three of the four populations defined by their expression of CD44 and CD25. The $CD44^-CD25^-$TN population is significantly greater in number ($P<0.05$) in males where there are almost twice the number present in females. In the $CD44^+CD25^-$, $CD44^+CD25^+$ and $CD44^-CD25^+$ TN populations there is no significant difference in the number of cells between male and female mice.

Comparison of the subsets defined by their expression of CD4 and CD8 at this age reveals no significant difference in the numbers of $CD4^-CD8^-$ cells ($P>0.05$) but double the number of $CD4^+CD8^+$ cells in female mice compared with male mice at this age (Figure 3ii). This was a significant increase ($P<0.01$). Increased numbers of cells in the female thymus was also noted at this time within the $CD4^+$ and $CD8^+$ single positive mature medullary thymocyte populations compared with the male thymus.

### Age changes in the female F5 transgenic mice

Analysis of the changes in number within the populations defined by their expression of CD4 and CD8 over the lifespan of the transgenic F5 female mouse is shown in Figure 4. As with the male F5 transgenic mouse there is none of the atrophy in the thymus seen with aging. Comparison of total cell number and number within the thymic subsets $CD4^-CD8^-$, $CD4^+CD8^+$, $CD4^-CD8^+$ at one time point with the same population at every other time point (ie at 3 months compared with 6.5, 9, 12 and 18 months, and at 6.5 months of age with those present at 9, 12 and 18 months, and at 9 months compared with 12 and 18 months, and at 12 months with 18 months of age) showed no significant difference (P was always greater than 0.05). Comparison of the numbers of cells in the major thymic subpopulations ($CD4^-CD8^-$, $CD4^+CD8^+$, $CD4^-CD8^+$) at 9 months of age for male and female transgenics are shown in Table I. In contrast to normal male and females at 9 months of age, there is no significant difference in numbers of $CD4^+CD8^+$ thymocytes.

TABLE I Analysis of thymocyte numbers defined by expression of CD4 and CD8 at 9 months of age in transgenic male and female mice (n=5 animals at each time point)

| *Population* | *Male* | *Female* | *P Value* |
|---|---|---|---|
| $CD4^-CD8^-$ | $10.7 \pm 2.6 \times 10^5$ | $14.2 \pm 4.4 \times 10^5$ | >0.05 |
| $CD4^+CD8^+$ | $5.7 \pm 1 \times 10^7$ | $7.8 \pm 1.7 \times 10^7$ | >0.05 |
| $CD4^-CD8^+$ | $15.4 \pm 2.2 \times 10^6$ | $2.4 \pm 1.3 \times 10^7$ | >0.05 |

### Comparison of CD3 expression at 9 months of age

Figure 5 shows expression of CD3 on $CD4^+CD8^+$ thymocytes on representative examples from male and female mice at 9 months of age and compares this with results from mice at 3 months of age. Analysis reveals that the $CD4^+CD8^+$ populations in male and female mice at 9 months of age show distinct differences in their fluorescent profiles. The $CD4^+CD8^+$ population in male and female mice at 9 months of age shows cells with bright CD3 staining but males contain fewer cells with the dull levels of CD3 expression seen in the female at this time. This experiment was repeated several times and the CD3 profile were similar in each sex showing the reproducibility of this gender difference (data not shown). The reduced dull CD3 population in the males at 9 months of age was linked to age and not sex as shown by the fluorescence profile in Figure 6. Analysis of the $CD4^+CD8^+$ population for expression of CD3 in normal male and female mice at 3 months of age is shown in this figure. The results reveal that anti-CD3 antibodies stain the gated $CD4^+CD8^+$ population producing a profile similar for both sexes at this age.

### DISCUSSION

This study clearly demonstrates gender-related difference in the rate at which the thymus atrophies with age. Thymic decline appears to be similar initially in both male and female mice but around 9 months of age there was a significantly higher number of cells in the female compared with the male thymus. It is unlikely that this could be accounted for by a single event in the maintenance of the animals since at this

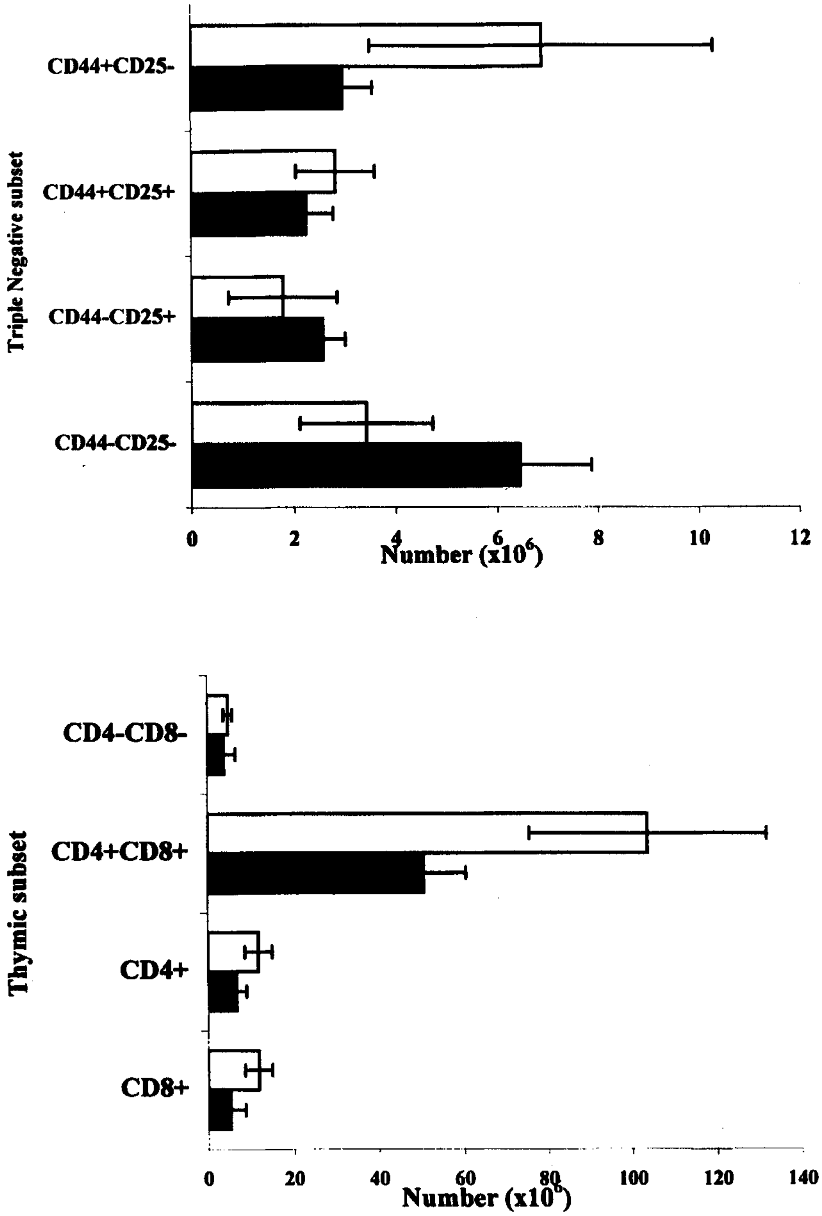

FIGURE 3 Analysis of thymocyte numbers at 9 months of age shows (i) similar numbers within all 4 TN populations defined by their expression of CD44 and CD25 and (ii) a significant increase ($P<0.01$) in the number of $CD4^{+}CD8^{+}$ cells in female (unfilled bars) mice when compared to males (filled bars). Increased numbers of $CD4^{+}$ and $CD8^{+}$ cells were noted in the female thymus at this time point ($n=4$)

time point animals were analysed in separate batches at time intervals which were months apart.

Previous work considered that the decline in the cellularity within the male thymus follows the principles of a single hit model (Aspinall, 1997). In this model a problem in the rearrangement of the TCRβ chain leads to a decline in the numbers of $CD44^{+}CD25^{+}$TN and their progeny the

$CD44^-CD25^+TN$ and $CD44^-CD25^-TN$ populations but no change in the numbers of their precursors the $CD44^+CD25^-TN$ population. The data comparing the numbers of cells at different stages in the T cell differentiation pathway prior to the expression of CD4, CD8 and CD3 would support the hypothesis that in females, as in males, there is a problem with the rearrangement of the TCR$\beta$ chain. This hypothesis is supported by results from F5 transgenic mice which do not show age-associated thymic atrophy. These mice carry a complete $\alpha\beta$TCR as a transgene (Mamalaki et al., 1993) and so have no requirement for rearrangement to produce a selectable TCR.

The results showing no significant difference between males and females in the numbers of $CD44^+CD25^-TN$, $CD44^+CD25^+TN$ and $CD44^-CD25^+$ TN populations at 9 months of age demonstrates that the factors affecting the production or survival of these cells are the same in both sexes. There were significant differences in the $CD44^-CD25^-$ TN population with almost double the number of these cells in males than females. The progeny of these cells are the $CD4^+CD8^+$ population and at 9 months of age there were significant differences in the number of these in females compared with males. These increased numbers in females produced from precursors whose numbers were less than the numbers of the same phenotypic population in males, indicates that there must be more permitted divisions within the $CD4^+CD8^+$ population in females compared with males.

A critical decision point within the $CD4^+CD8^+$population is positive selection on the basis of the $\alpha\beta$TCR pairing achieved (Jameson et al., 1995). This is critical because it impacts upon the diversity of the potential repertoire to be generated. Because of this it was important to determine whether the gender differences in $CD4^+CD8^+$ cell numbers represent differences prior to positive selection or after positive selection. The data from normal animals showing more $CD4^+CD8^+$ cells with higher expression of CD3 at 9 months of age in females compared with males are not conclusive on this issue. However the results from the transgenic F5 mice are conclusive and indicate that the differences seen in the normal mice must be due to greater division before positive selection.

Intrathymic T cell differentiation in the F5 mice is normal, proceeding through the same developmental stages as non-transgenic mice. F5 mice show the same changes in the levels of TCR expressed at different stages of thymocyte development, with increased expression correlating with maturity of the thymic subpopulation. In addition the thymocytes in F5 mice show positive selection in C57BL/10 mice and negative selection in animals expressing Class II MHC IE molecules and the endogenous Mtv ligand (Mamalaki et al., 1993).

The F5 data indicates that the difference seen in normal animals is not due to clonal expansion after production of a functional $\alpha\beta$TCR, since this would result in an increased number of $CD4^+CD8^+$ cells in F5 females compared with males, a result which was not observed. The differences must be due to gender related differences in the number of permitted divisions prior to positive selection at 9 months of age.

Any proposal that gender differences in the $CD4^+CD8^+$population represent differences in the transit time through these subpopulations would also be difficult to sustain in view of the result from the transgenic mice.

The rate of thymic atrophy between males and females appears to be similar between the ages of 3 and 6 months but differences arise at 9 months of age when thymic size is greater in females than in males. This movement of the female thymus away from the trend of decline seen in the male may be the result of some stimulatory effect in the female or some inhibitory effect in the male. Explanations of these gender differences include both hormonal causes and immune related mechanisms. For a hormonal related mechanism, testosterone would seem to provide a solution to the difference since it is known to induce apoptosis in $CD4^+CD8^+$ cells (Olsen et al., 1998). Furthermore reduction in testosterone levels by orchidectomy leads to a reversal of age related thymic atrophy and an increase in thymic subpopulations, which could be inhibited by testosterone in a dose dependant fashion (Greenstein et al., 1986). But the lack of any significant difference in the numbers of cells between male and female F5 transgenic mice makes this hypothesis difficult to sustain. The hypoth-

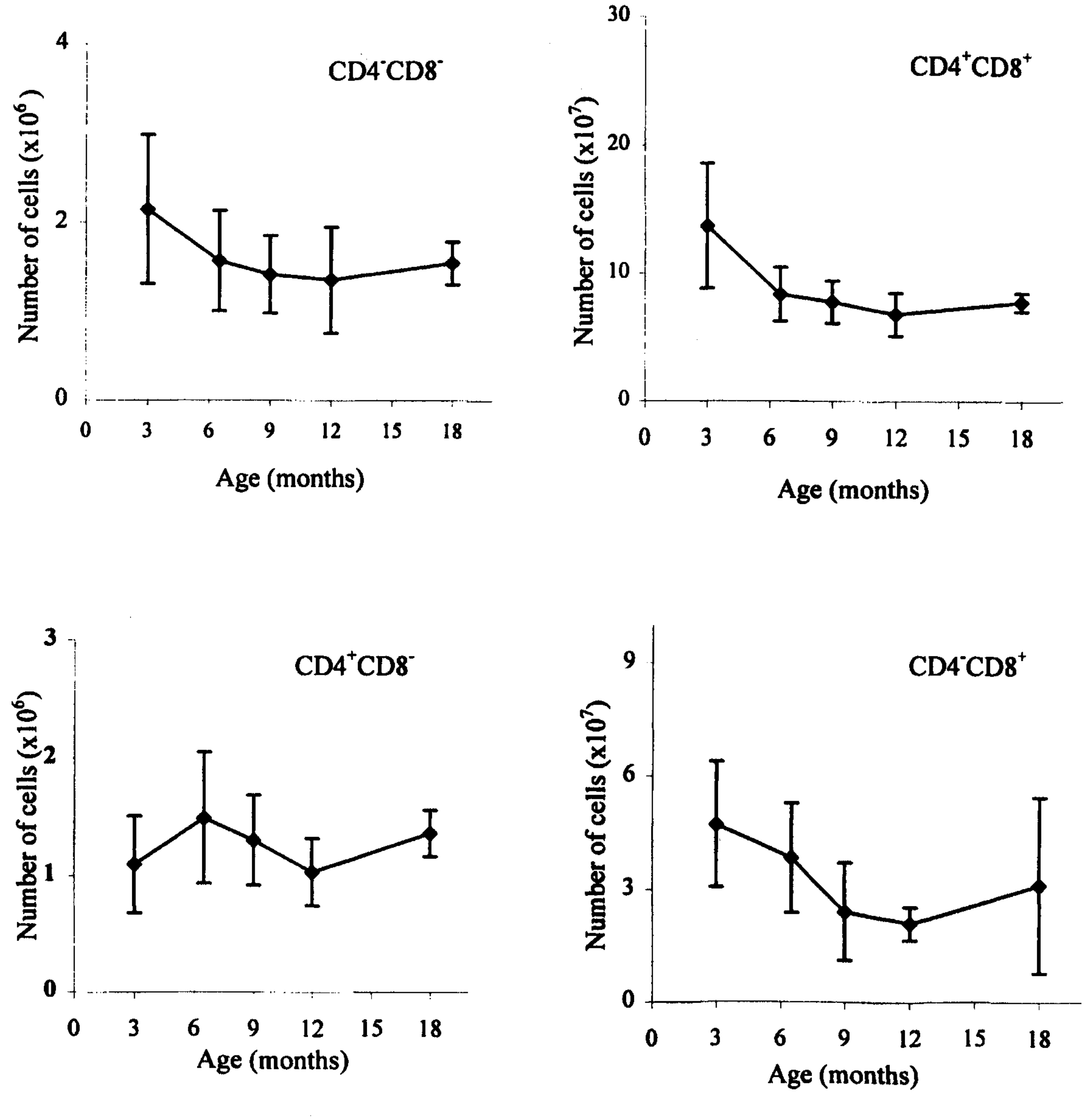

FIGURE 4 Age associated thymic atrophy was not observed in female F5 transgenic mice. Comparison of the numbers within the population defined by their expression of CD4 and CD8 in female mice reveals no difference with age (n=3–6 animals at each time point)

esis would only be tenable if the F5 were not susceptible to the effects of testosterone. Experiments carried out to test this hypothesis revealed that cultures of fetal F5 thymuses in the presence of different concentrations of testosterone demonstrated similar reductions in thymic subpopulations as seen with fetal thymuses from normal animals (data not shown).

Alternatively the differences may be due to alterations in the levels of oestrogen with age, since oestrogen is also known to inhibit thymocyte development

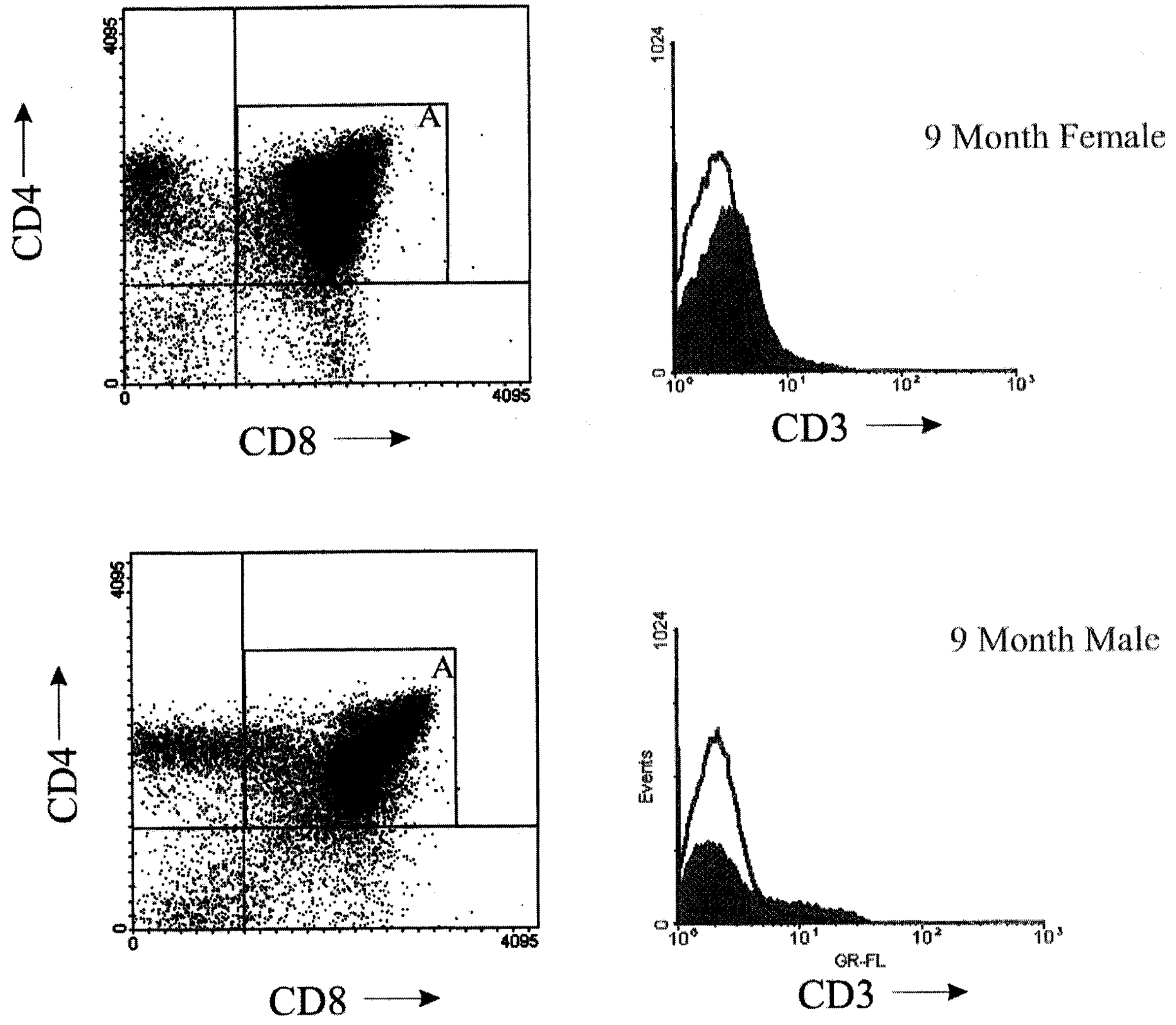

FIGURE 5 A representative experiment demonstrating low to intermediate expression of CD3 on $CD4^+CD8^+$ thymocytes is markedly reduced in males but not in females by 9 months of age

(Rijhsinghani et al., 1997). The idea that a rapid age related reduction in the levels of oestrogen produced less inhibition allowing more permitted divisions at 9 months of age in female $CD4^+CD8^+$ cells rather than males seems unlikely following previous studies showing that oophorectomy does not enhance T cell development (Rijhsinghani et al., 1996).

A potential mechanism to account for the gender related differences relates to a regulatory feedback pathway in the thymus. Previous reports suggested that there was negative feedback control over the total production of lymphocytes by the thymus acting at the $CD4^+CD8^+$ stage of the pathway (Mehr et al., 1996). The mechanism involves the effect of mature $CD4^+$ T cells acting on these stages through the cytokines they produce (Mehr et al., 1997). Gender differences have been shown to affect the cytokine production by T cells following antigen challenge

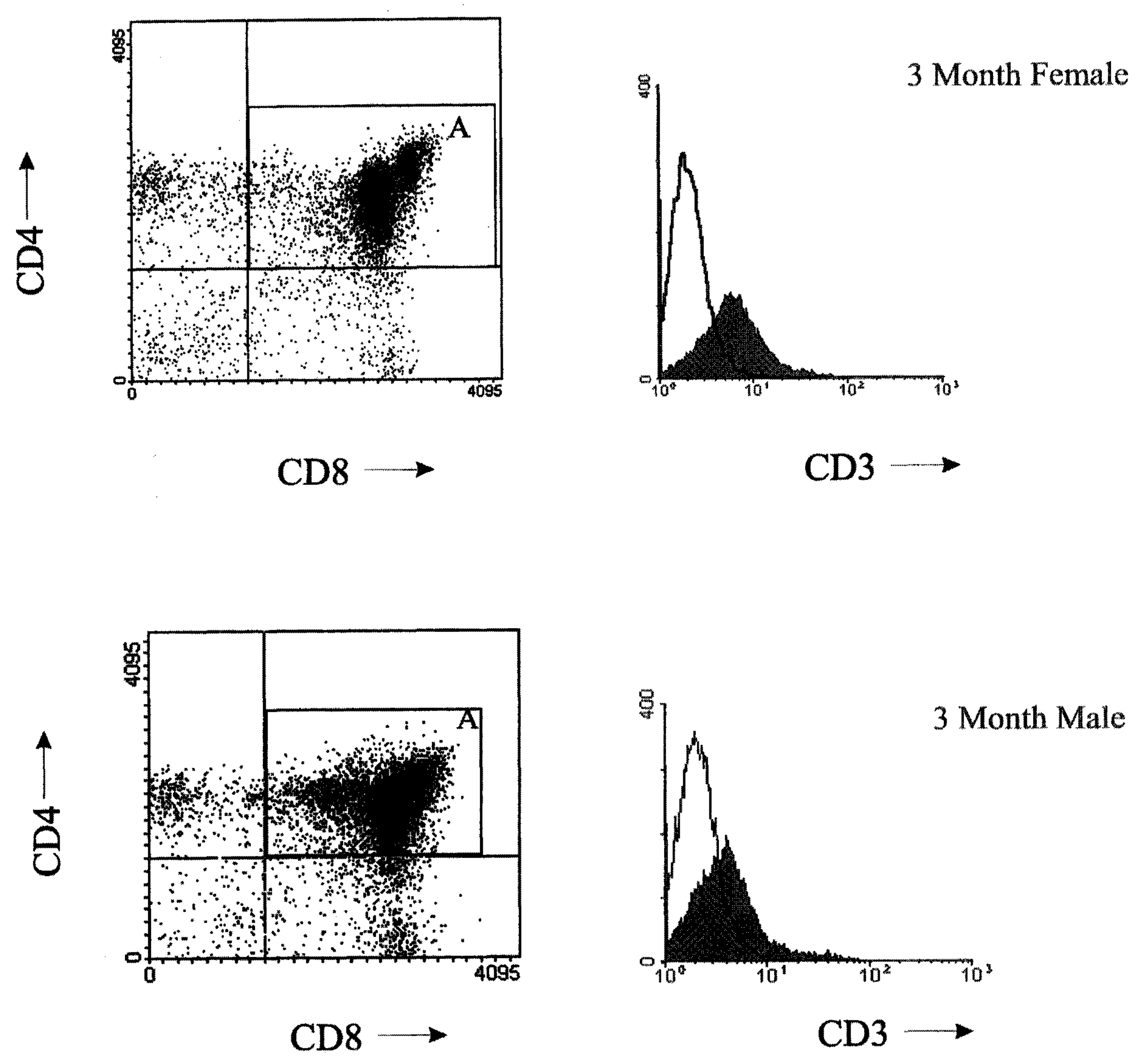

FIGURE 6 A representative experiment demonstrating similar CD3 expression on $CD4^{+}CD8^{+}$ thymocytes in males and females at 3 months of age thus having an influence on the $T_H1/T_H2$ balance depending on the hormone and dose (Piccinni et al., 1995; Dalal et al., 1997; Whitacre et al., 1999). Since recirculation from the periphery back to the thymus is restricted to activated T cells (Surh et al., 1993; Agus et al., 1991), the cells entering the thymus at 9 months of age will be secreting cytokines which in turn may alter the maturation kinetics of the $CD4^{+}CD8^{+}$ population. However this effect is not permanent since in normal mice the differences do not exist after 9 months of age. An alternative explanation is that there are differences in the numbers of dendritic cells entering the thymus in males and female mice at this age, since there may be a correlation between the number of dendritic cells and the number of $CD4^+CD8^+$cells (Anderson et al., 1998). However why such an effect is transitory is difficult to envisage.

The conclusions then from this work is that age associated thymic atrophy in females is due to a problem with rearrangement of the TCRβ chain. This is shown by female F5 transgenic mice failing to undergo age-associated thymic atrophy, which is identical to that seen with males. Furthermore in normal non-transgenic male and female mice it is clear that the lowest point of thymic atrophy is reached by 12 months of age after which time there appear to be no sex – related differences in thymic size. Before this age however, the differences seen in the thymus between normal male and female animals would suggest that there is an additional cause of atrophy in the male that permits fewer divisions between the phenotype stages of $CD44^-CD25^-$ TN and $CD4^+CD8^+$. This would seem to show the greatest difference at 9 months of age. At present the most likely explanation for this is a negative feedback due to mature $CD4^+$ T cells affecting this stage of the pathway.

The implications of this are that males of this age will show fewer TCRαβ pairings that in turn may restrict the potential repertoire in males later in life. This also implies that the female mice should have a larger potential repertoire later in life than males.

### Acknowledgements

We are grateful to Professor Frances Gotch and Dr Nesrina Imami for their comments on the manuscript and for fruitful discussions on this work. The work was supported by the Wellcome Trust (Grant Number 051541).

### References

(1992). The Government Actuary's Department.

(1997). Office of National Statistics. (Abstract).

Agus, D.B., Surh, C.D., and Sprent, J. (1991). Reentry of T cells to the adult thymus is restricted to activated T cells. J. Exp. Med. **173**, 1039–1046.

Alam, S.M., Travers, P.J., Wung, J.L., Nasholds, W., Redpath, S., Jameson, S.C., and Gascoigne, N.R. (1996). T-cell-receptor affinity and thymocyte positive selection [see comments]. Nature **381**, 616–620.

Anderson, G., Partington, K.M., and Jenkinson, E.J. (1998). Differential effects of peptide diversity and stromal cell type in positive and negative selection in the thymus. J. Immunol. **161**, 6599–6603.

Aspinall, R. (1997). Age-associated thymic atrophy in the mouse is due to a deficiency affecting rearrangement of the TCR during intrathymic T cell development. J. Immunol. **158**, 3037–3045.

Bloom, E.T. and Horvath, J.A. (1994). Cellular and molecular mechanisms of the IL-12-induced increase in allospecific murine cytolytic T cell activity. Implications for the age-related decline in CTL. J. Immunol. **152**, 4242–4254.

Brandle, D., Muller, C., Rulicke, T., Hengartner, H., and Pircher, H. (1992). Engagement of the T-cell receptor during positive selection in the thymus down-regulates RAG-1 expression. Proc. Natl. Acad. Sci. U.S.A. **89**, 9529–9533.

Dalal, M., Kim, S., and Voskuhl, R.R. (1997). Testosterone therapy ameliorates experimental autoimmune encephalomyelitis and induces a T helper 2 bias in the autoantigen-specific T lymphocyte response. J. Immunol. **159**, 3–6.

Flurkey, K., Miller, R.A., and Harrison, D.E. (1992). Cellular determinants of age-related decrements in the T-cell mitogen response of B6CBAF1 mice. J. Gerontol. **47**, B115–20.

Gardner, I.D. (1980). The effect of aging on susceptibility to infection. Rev. Infect. Dis. **2**, 801–810.

Godfrey, D.I., Kennedy, J., Suda, T., and Zlotnik, A. (1993). A developmental pathway involving four phenotypically and functionally distinct subsets of CD3-CD4-CD8- triple-negative adult mouse thymocytes defined by CD44 and CD25 expression. J. Immunol. **150**, 4244–4252.

Godfrey, D.I., Kennedy, J., Mombaerts, P., Tonegawa, S., and Zlotnik, A. (1994). Onset of TCR-beta gene rearrangement and role of TCR-beta expression during CD3-CD4-CD8- thymocyte differentiation. J. Immunol. **152**, 4783–4792.

Greenstein, B.D., Fitzpatrick, F.T., Adcock, I.M., Kendall, M.D., and Wheeler, M.J. (1986). Reappearance of the thymus in old rats after orchidectomy: inhibition of regeneration by testosterone. J. Endocrinol. **110**, 417–422.

Hannet, I., Erkeller Yuksel, F., Lydyard, P., Deneys, V., and DeBruyere, M. (1992). Developmental and maturational changes in human blood lymphocyte subpopulations [see comments]. Immunol. Today **13**, 215,218.

Hulstaert, F., Hannet, I., Deneys, V., Munhyeshuli, V., Reichert, T., De Bruyere, M., and Strauss, K. (1994). Age-related changes in human blood lymphocyte subpopulations. II. Varying kinetics of percentage and absolute count measurements Age-related changes in human blood lymphocyte subpopulations. II. Varying kinetics of percentage and absolute count measurements. Clin. Immunol. Immunopathol. **70**, 152–158.

Jameson, S.C., Hogquist, K.A., and Bevan, M.J. (1995). Positive selection of thymocytes. Annu. Rev. Immunol. **13**, 93–126.

Mackall, C.L., Fleisher, T.A., Brown, M.R., Andrich, M.P., Chen, C.C., Feuerstein, I.M., Horowitz, M.E., Magrath, I.T., Shad, A.T., Steinberg, S.M., and et al (1995). Age, thymopoiesis, and CD4+ T-lymphocyte regeneration after intensive chemotherapy [see comments]. N. Engl. J. Med. **332**, 143–149.

Mackall, C.L. and Gress, R.E. (1997). Thymic aging and T-cell regeneration. Immunol. Rev. **160**, 91–102.

Mamalaki, C., Elliott, J., Norton, T., Yannoutsos, N., Townsend, A.R., Chandler, P., Simpson, E., and Kioussis, D. (1993). Positive and negative selection in transgenic mice expressing a T-cell receptor specific for influenza nucleoprotein and endogenous superantigen. Dev. Immunol. **3**, 159–174.

Mehr, R., Perelson, A.S., Fridkis Hareli, M., and Globerson, A. (1996). Feedback regulation of T cell development: manifestations in aging. Mech. Ageing Dev. **91**, 195–210.

Mehr, R., Perelson, A.S., Fridkis Hareli, M., and Globerson, A. (1997). Regulatory feedback pathways in the thymus. Immunol. Today **18**, 581–585.

Olsen, N.J., Viselli, S.M., Fan, J., and Kovacs, W.J. (1998). Androgens accelerate thymocyte apoptosis. Endocrinology **139**, 748–752.

Penit, C. and Vasseur, F. (1997). Expansion of mature thymocyte subsets before emigration to the periphery. J. Immunol. **159**, 4848–4856.

Petrie, H.T., Livak, F., Schatz, D.G., Strasser, A., Crispe, I.N., and Shortman, K. (1993). Multiple rearrangements in T cell receptor alpha chain genes maximize the production of useful thymocytes. J. Exp. Med. **178**, 615–622.

Piccinni, M.P., Giudizi, M.G., Biagiotti, R., Beloni, L., Giannarini, L., Sampognaro, S., Parronchi, P., Manetti, R., Annunziato, F., Livi, C., and et al (1995). Progesterone favors the development of human T helper cells producing Th2-type cytokines and promotes both IL-4 production and membrane CD30 expression in established Th1 cell clones. J. Immunol. **155**, 128–133.

Rijhsinghani, A.G., Thompson, K., Bhatia, S.K., and Waldschmidt, T.J. (1996). Estrogen blocks early T cell development in the thymus. Am. J. Reprod. Immunol. **36**, 269–277.

Rijhsinghani, A., Bhatia, S.K., Kantamneni, L., Schlueter, A., and Waldschmidt, T.J. (1997). Estrogen inhibits fetal thymocyte development in vitro. Am. J. Reprod. Immunol. **37**, 384–390.

Simpson, J.G., Gray, E.S., and Beck, J.S. (1975). Age involution in the normal human adult thymus. Clin. Exp. Immunol. **19**, 261–265.

Smith, S.M. and Ossa Gomez, L.J. (1981). A quantitative histologic comparison of the thymus in 100 healthy and diseased adults. Am. J. Clin. Pathol. **76**, 657–665.

Steinmann, G.G. (1986). Changes in the human thymus during aging. Curr. Top. Pathol. **75**, 43–88.

Surh, C.D., Sprent, J., and Webb, S.R. (1993). Exclusion of circulating T cells from the thymus does not apply in the neonatal period. J. Exp. Med. **177**, 379–385.

Whitacre, C.A., Reingold, S.C., O'Looney, P.A., and Task Force (1999). A Gender Gap in Autoimmunity. Scince **283**, 1277–1278.

Wu, L., Scollay, R., Egerton, M., Pearse, M., Spangrude, G.J., and Shortman, K. (1991). CD4 expressed on earliest T-lineage precursor cells in the adult murine thymus. Nature **349**, 71–74.

